# Diffuse duodenal nodular lymphoid hyperplasia: a large cohort of patients etiologically related to *Helicobacter pylori *infection

**DOI:** 10.1186/1471-230X-11-36

**Published:** 2011-04-11

**Authors:** Mehnaaz S Khuroo, Naira S Khuroo, Mohammad S Khuroo

**Affiliations:** 1 Lecturer, Department of Pathology, Government Medical college, Srinagar, Kashmir, India; 2 Consultant Radiology, Digestive Diseases Centre, Srinagar, Kashmir, India; 3 Consultant Gastroenterology, Digestive Diseases Centre, Srinagar, Kashmir, India

## Abstract

**Background:**

Nodular lymphoid hyperplasia of gastrointestinal tract is a rare disorder, often associated with immunodeficiency syndromes. There are no published reports of its association with *Helicobacter pylori *infection.

**Methods:**

From March 2005 till February 2010, we prospectively followed all patients with diffuse duodenal nodular lymphoid hyperplasia (DDNLH). Patients underwent esophagogastroduodenoscopy with targeted biopsies, colonoscopy, and small bowel video capsule endoscopy. Duodenal nodular lesions were graded from 0 to 4 based on their size and density. Patients were screened for celiac sprue (IgA endomysial antibody), immunoglobulin abnormalities (immunoglobulin levels & serum protein electrophoresis), small intestine bacterial overgrowth (lactulose hydrogen breath test), and *Helicobacter pylori *infection (rapid urease test, and histological examination of gastric biopsies). Patients infected with *Helicobacter pylori *received sequential antibiotic therapy and eradication of infection was evaluated by ^14^C urea breath test. Follow up duodenoscopies with biopsies were performed to ascertain resolution of nodular lesions.

**Results:**

Forty patients (Males 23, females 17; mean age ± 1SD 35.6 ± 14.6 years) with DDNLH were studied. Patients presented with epigastric pain, vomiting, and weight loss. Esophagogastroduodenoscopy showed diffuse nodular lesions (size varying from 2 to 5 mm or more) of varying grades (mean score ± 1SD 2.70 ± 0.84) involving postbulbar duodenum. Video capsule endoscopies revealed nodular disease exclusively limited to duodenum. None of the patients had immunoglobulin deficiency or small intestine bacterial overgrowth or positive IgA endomysial antibodies. All patients were infected with *Helicobacter pylori *infection. Sequential antibiotic therapy eradicated *Helicobacter pylori *infection in 26 patients. Follow up duodenoscopies in these patients showed significant reduction of duodenal nodular lesions score (2.69 ± 0.79 to 1.50 ± 1.10; p < 0.001). Nodular lesions showed complete resolution in 5 patients and significant resolution in remaining 21 patients. Patients with resistant *Helicobacter pylori *infection showed no significant reduction of nodular lesions score (2.71 ± 0.96 to 2.64 ± 1.15; p = 0.58). Nodules partially regressed in score in 2 patients, showed no interval change in 10 patients and progressed in 2 patients.

**Conclusions:**

We report on a large cohort of patients with DDNLH, etiologically related to *Helicobacter pylori *infection.

## Background

Nodular lymphoid hyperplasia (NLH) of the gastrointestinal tract represents a rare disease that is grossly characterized by the presence of numerous visible mucosal nodules measuring up to, and rarely exceeding, 0.5 cm in diameter [1]. Histologically, hyperplasic lymphoid follicles with large germinal centres are seen in the lamina propria and superficial submucosa [2]. There is enlargement of the mucosal B cell follicles caused by hyperplasia of the follicle centres; surrounded by a normal appearing mantle zone. Disease may involve the stomach, the entire small intestine, and the large intestine [3]. NLH involving the colon can mimic a variety of polyposis syndromes and this may cause difficulties in diagnosis [4]. Disease has been reported to cause pulmonary disease as well [5]. The etiology is unknown. In children, NLH is often associated with viral infection or food allergy; tends to have a benign course and usually regresses spontaneously [6,7]. The disease in adults is rare and poorly described [8]. It has been suggested that NLH is a risk factor for both intestinal and extra intestinal lymphoma [9-11]. Approximately 20% of adults with common variable immunodeficiency are found to have NLH [12]. Some patients have low or absent IgA and IgM levels, decreased IgG levels, susceptibility to infection, small intestine bacterial overgrowth, diarrhea with or without steatorrhea [13-16]. *Giardia lamblia *is often present in such patients [17-19]. There is also an association with familial adenomatous polyposis and Gardner's syndrome [20]. It has also been reported in patients with human immunodeficiency virus infection [21]. The disease may be associated with other pathologies, especially gastrointestinal malignancies [22]. Except an isolated case of gastric nodular lymphoid hyperplasia, there are no published reports of association of NLH with *Helicobacter pylori *(*H. pylori*) infection [23]. Here, we report on a large cohort of patients with NLH, etiologically related to *H. pylori *infection.

## Methods

### Study Protocol

From March 2005 till February 2010, we prospectively followed all patients with diffuse duodenal nodular lymphoid hyperplasia (DDNLH). Patients had detailed history and physical examination. Complete blood counts and serum chemistry were done by standard techniques. Stool analysis was done for ova and parasites. Giardia lamblia infection was evaluated by examinations of concentrated, iodine-stained wet stool preparations; duodenal aspirates and duodenal biopsies. IgA endomysial antibodies were detected by indirect immunofluorescence assay. Serum immunoglobulin (IgG, IgA & IgM) were estimated by immunoturbidometry. Serum protein electrophoresis was performed by agarose gel electrophoresis and densitometry. Small intestine bacterial overgrowth was evaluated by lactulose hydrogen breath test. Patient underwent esophagogastroduodenoscopy (EGD), targeted gastric and duodenal biopsies, evaluation of *H. pylori *infection; colonoscopies with ileoscopy and video capsule endoscopy. Patients infected with *H. pylori *received 10 days sequential antibiotic therapy. Eradication of *H. pylori *was evaluated by ^14^C urea breath Test (^14^C UBT) 4 to 6 weeks after antibiotic therapy. Patients resistant to sequential therapy received second line antibiotic therapy. Follow up EGD's were performed at/after 6 months of antibiotic therapy to assess the status of the duodenal nodular lesions detected earlier.

### Diffuse Duodenal Nodular Lymphoid Hyperplasia (DDNLH)

Nodular lymphoid hyperplasia was diagnosed when numerous mucosal nodules (2 to 5 mm or more) were visible on endoscopic examination of the gut mucosa and histological examination of the forceps punch biopsies from nodules was reported as NLH [1]. NLH was characterized by presence of well-circumscribed nodes of lymphoid tissue in the lamina propria and/or superficial submucosa. These lymphoid collections showed presence of highly reactive germinal centres, numerous cell types, prominent vascularity and polyclonality as determined immunohistochemically [2]. The extent of nodular lesions was evaluated by various imaging tools including esophagogastroduodenoscopy, colonoscopy with ileoscopy and video capsule endoscopy. Diffuse nodular lesions limited to duodenum were diagnosed as DDNLH.

### Esophagogastroduodenoscopy (EGD)

EGD was performed by an experienced endoscopist (MSK 3) with video-esophago-gastro-duodenoscope (Olympus Evis Smartage Gastro GIF V70 Serial, Olympus Japan). Procedures were video-recorded and representative findings documented on high resolution images using software program. Endoscopic findings were recorded on a proforma. Duodenum was carefully examined for nodules (circumscribed elevated mucosal lesions varying in size from 2 mm to 5 mm or more). Nodular lesions in the postbulbar duodenum, second and third part were graded on a scale of 0 to 4, depending upon the size and density of nodules. Grading was done by 2 investigators (MSK 3/NSK), blinded to results, by reviewing EGD images and recorded video. Prior to actual grading, the scoring system was mutually agreed upon and any discrepancies were mutually discussed and sorted out. Normal duodenum (grade 0) showed prominent smooth closely spaced *Valvulae conniventes *(*Kerckring's folds*) without scalloping or nodules. Nodules around 2 to 3 mm in size and less than 20 in number in the region inspected were reported as grade1. Similar size numerous (>20) nodules which had not deformed the duodenal folds were reported as grade 2. Grade 3 nodular disease was seen as numerous large (2 to 5 mm) nodules carpeting the mucosa and causing mucosal fold invasion and deformity. Nodules which were more than 5 mm in size carpeting the whole duodenal mucosa and obscuring the mucosal folds were reported as grade 4. Multiple (6 or more) forceps biopsies were taken from nodules and the intervening mucosa for histology. Biopsy specimens were examined under light microscopy after staining with hematoxylin and eosin; Periodic Acid Schiff (PAS) and reticulin stains. Immunohistochemistry using CD3 & CD20 markers was performed to evaluate the cell (T & B cells) population in the lymphoid nodules. Biopsies were examined for viral inclusions and parasites especially *Giardia lamblia *[24]. *H. pylori *infection was evaluated by rapid urease test (RUT) and at histology. For RUT, 2 forceps punch biopsies were taken from gastric incisura and embedded in agar gel urea-rich medium (HP test™, Allied Marketing Corporation, Kolkata, India) and read as per manufacturer's instructions. Multiple gastric biopsies (two from antrum; two from body and additional specimens from any visible endoscopic visible lesions, if needed) were taken and stained with Hematoxylin & Eosin to type and grade gastritis; Alcian blue to detect intestinal metaplasia and Giemsa stain for *H. pylori *detection and density [25,26].

### Colonoscopy

Colonoscopy with ileoscopy was performed by an experienced endoscopist (MSK) with video-colonoscope (Olympus Evis Smartage Colono CF V70L Serial, Olympus Japan). Forceps biopsies were taken from endoscopic abnormalities as well as terminal ileum.

### Video Capsule Endoscopy

Small bowel video capsule endoscopy was performed on capsule endoscopy system, Rapid 5 UPG with help of Pillcam SB2 capsule (Given Imaging, Ltd. Israel). Video images were examined for presence and extent of nodular lesions in the small bowel.

### Lactulose Hydrogen Breath Test

Small intestinal bacterial overgrowth was evaluated by analyzing breath hydrogen with Gastrolyzer (Bedfont Scientific Ltd. Rochester, Kent, England) after a challenge dose of lactulose (10 g) and breath samples collected at baseline and subsequent 20 minutes intervals for 3 hours. Positive diagnosis for small intestine bacterial overgrowth was made if the breath hydrogen showed 20 ppm rise above baseline within first 2 hours [27].

### *H. pylori *Eradication

*H. pylori *eradication was done with sequential antibiotic therapy. Patients received pantoprazole, 40 mg, with amoxillin, 1 g, twice daily for 5 days followed by pantoprazole, 40 mg, clarithromycin, 500 mg, and tinidazole, 500 mg, twice daily for next five days [28]. Following sequential therapy, patients received symptomatic treatment including pantoprazole 40 mg per day for next 4 weeks. Patients with resistant *H. pylori *infection to sequential therapy received 10 days second line therapy (pantoprazole 40 mg twice daily along with levofloxacin 750 mg and doxycycline 100 mg once daily). This antibiotic combination was based on *H. pylori *antibiotic sensitivity in this region (Khuroo et al unpublished data).

### ^14^C Urea Breath Test (^14^C UBT)

Eradication of *H. pylori *infection after antibiotic therapy was evaluated by ^14^C UBT using Heliprobe™ System 2000 (Kibion AB, Uppsala, Sweden). After an overnight fast, patient swallowed a ^14^C Urea Capsule (HeliCap™). After 10 minute wait, patient breathed in to a breath card (BreathCard™) till acid sensitive indicator changed color from orange to yellow suggesting adequate trapping of exhaled breath ^14^Carbon dioxide as lithium carbonate. Breath Card ^14^C activity was analyzed in a Geiger Muller Counter (Probe™) and *H. pylori *infection analyzed and presented on the display as Heliprobe TM grade 0 (not infected), 1 (Indeterminate) and 2 (Infected).

### Ethics

Written informed consent explaining the indications, adverse affects and alternatives was obtained from all patients before the procedures were carried out. The study protocol was submitted to the ethical committee of Digestive Diseases Centre and was approved. The study protocol conformed to good medical practice as defined in the Helsinki principles.

### Statistics

Comparisons of the categorical variables were analyzed using the Fisher's exact test. Comparisons of the continuous variables were analyzed using the Student's t-test. All values are expressed as mean ± SD and frequencies. The statistical analysis was carried out using SPSS for Windows (version 13.0). Two-tailed P values less than 0.05 were considered significant.

## Results

From March 2005 till February 2010, 44 patients with NLH of gastrointestinal tract were diagnosed. Two patients had disease limited to distal ileum as visualized at colonoscopy & ileoscopy. Another 2 patients had extensive disease involving duodenum, jejunum and ileum. These 2 patients had common variable immunodeficiency syndrome and one of them presented with superadded recurrent *Giardia lamblia *infection, Remaining 40 patients (Males 23, females 17; mean age ± 1SD 35.6 ± 14.6 years; age range 14 to 62 years) had diffuse nodular disease limited to duodenum and formed the study group (Table 1).

**Table 1:** The clinical profile of 40 patients with diffuse duodenal nodular hyperplasia.

Group	All patients	H pylori eradicated group	H pylori persistent group	P value (Eradicated vs. Non-eradicated)
Number of patients	40	26	14	
Age (years ± 1SD)	35.6 ± 14.6	33.6 ± 11.6	37.6 ± 15.6	0.29c
Sex (Male: Female)	23:17	15:11	8:6	0.64a
Symptoms (Number of patients)				
Pain	40	26	14	1.00a
Vomiting	38	24	14	0.41a
Weight loss	35	23	12	0.77a
Diarrhoea	6	4	2	0.65a
Constipation	4	1	3	0.11a
Hemoglobin (g/dl)	9.5 ± 2.2	8.9 ± 3.2	9.7 ± 1.2	0.33c
Serum albumin (g/dl)	3.2 ± 1.1	3.4 ± 1.1	2.9 ± 1.4	0.39c
Immunoglobulins (Number of patients)				
Normal	26	18	8	0.14a
Elevated IgG	6	4	2	0.65a
Elevated IgM	5	4	1	0.41a
Elevated IgA	3	0	3	0.03a
Gastroscopic findings (number of patients)				
No abnormality	8	6	2	0.41a
Antral gastritis	20	11	9	0.16a
Fundic exudative gastritis	6	4	2	0.65a
Atrophic gastritis	4	3	1	0.56a
Ulcerative Nodular antral disease	2	2	0	0.41a
Grade of duodenal nodular disease (Number of patients)				
Grade 1	4	2	2	0.71b
Grade 2	10	7	3	
Grade 3	20	14	6	
Grade 4	6	3	3	
Mean ± 1SD Score	2.70 ± 0.84	2.69 ± 0.79	2.71 ± 0.96	0.94c
Follow up months (mean ± 1SD)	24 ± 12.5 (6-56)	22 ± 14.5 (6-54)	26 ± 12.9 (8-56)	0.65c

a = Fisher's exact test 2 × 2 table; b = Fisher's exact test rxc table; c = students t test.

Key: The profile of 26 patients in whom *H. pylori *infection was eradicated and 14 patients in whom *H. pylori *infection was resistant to eradication therapy are compared.

Dominant clinical presentation was epigastric pain, postprandial abdominal distension, vomiting and weight loss. Duration of symptoms varied from 6 months to 5 years (mean ± 1 SD 2.6 ± 1.2 years). Six patients presented with recurrent episodes of diarrhoea, while 4 patients complained of constipation. None of the patients had history suggestive of steatorrhea. Hemoglobin ranged from 6 g/dl to 12.5 g/dl (mean ± 1SD 9.5 ± 2.2 g/dl). Six patients had severe anemia (<6.0 g/dl), 22 patients had moderate anemia (6 to <8 g/dl) and 12 patients had mild anemia (8 to <10 g/dl). Haematological indices revealed iron deficiency anemia in all patients. None of the patients had megaloblastic anemia. Serum albumin ranged from 2.5 g/dl to 4.5 g/dl (mean ± 1SD 3.2 ± 1.1 g/dl). 8 patients had low serum albumin. Serum calcium levels were below normal in 10 patients. Immunoglobulin levels were within normal limits in 26 patients. 14 patients had elevated immunoglobulin levels (IgG in 6 patients, IgM in 5 patients, and IgA in 3 patients). Elevated immunoglobulin levels were less than 1.5 times of the upper limit of normal values. None of the patients had low immunoglobulin level. Serum protein electrophoresis showed normal electrophoretic pattern and none of the patients had M band pattern. None of the patients had small intestine bacterial overgrowth as assessed by lactulose hydrogen breath test. IgA endomysial antibodies were negative in all patients. Stool analysis revealed ova of *Ascaris lumbricoides *in 11 patients. All patients with *Ascaris lumbricoides *infection received anthelmintic therapy namely mebendazole 100 mg twice daily per oral for 3 days. None of the patients had *Giardia lamblia *infection *as *evaluated by examinations of concentrated, iodine-stained wet stool preparations; duodenal aspirates and duodenal biopsies.

**Figure 1:**
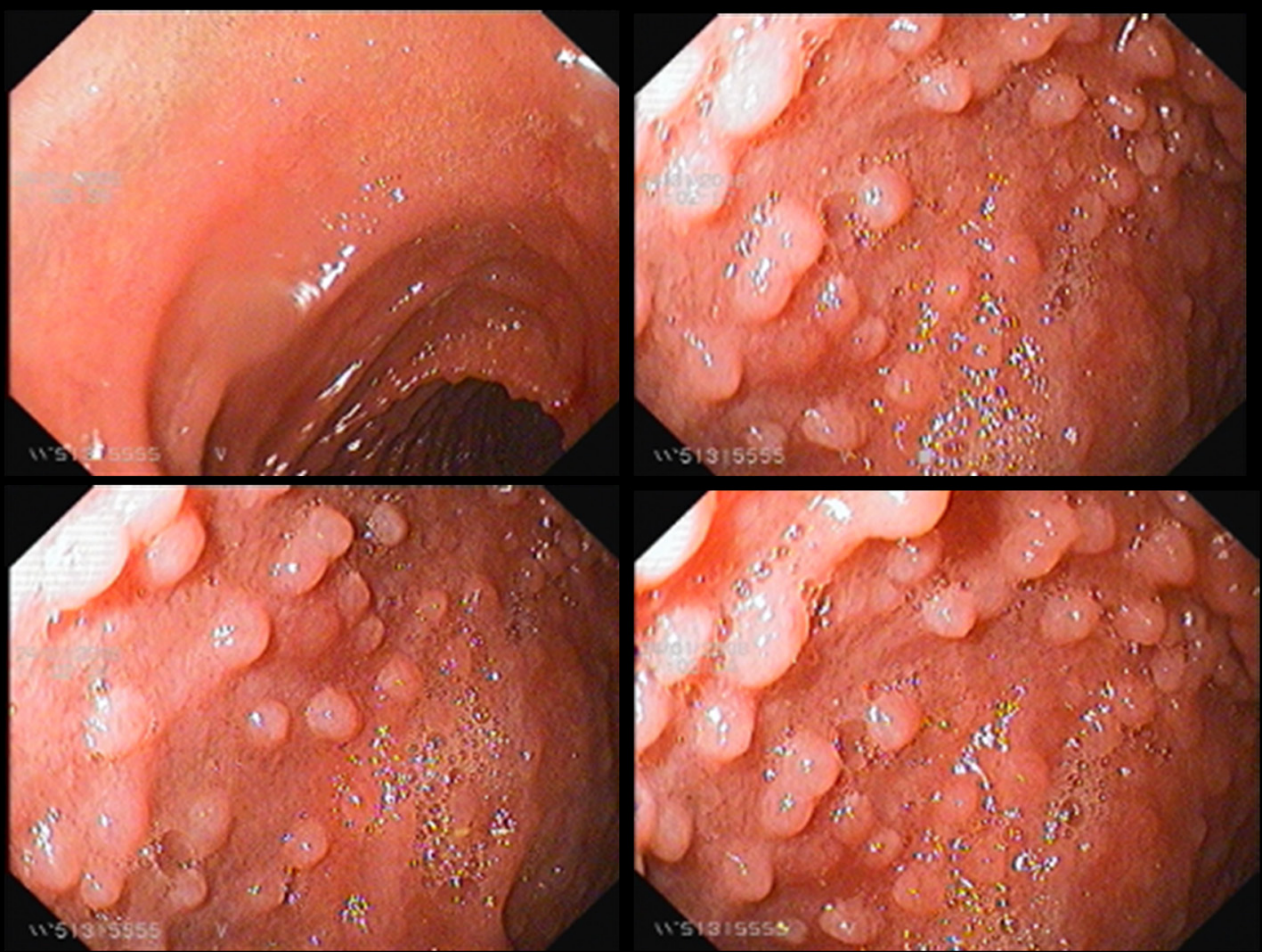
**Diffuse Duodenal Nodular Lymphoid Hyperplasia**. 35 year male presented with epigastric pain, vomiting and weight loss of 5 kg over the past 6 months. Duodenal composite images. Top left image: Duodenal bulb revealed smooth mucosa without any nodular lesion. Top right and 2 bottom images: post-bulbar region, second and third part of duodenum showed diffuse numerous mucosal nodules (>5 mm size each) scored as grade 4 disease. There was complete loss of *Kerckring's folds*. Duodenal biopsies revealed nodular lymphoid hyperplasia. Immunoglobulin revealed mild elevation of IgG level [IgG 2090 mg/dl (normal 700-1600 mg/dl); IgM. 120 mg/dl (normal = 40-230 mg/dl); IgA 145 mg/dl (normal = 70-400 mg/dl)].

**Figure 2:**
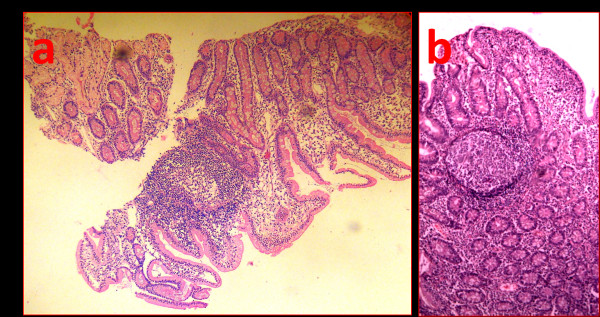
**Diffuse Duodenal Nodular Lymphoid Hyperplasia**. a) Low power view (H&E 10×) of duodenal biopsy showing a lymphoid follicle. b) High power view (H&E 40×) A mature lymphoid follicle with highly reactive germinal centre and surrounding normal appearing mantle zone is seen. There is mild increase in lymphoid infiltrate in the lamina propria.

EGD showed diffuse nodular duodenal lesions in all patients (Figure 1). Duodenal bulb showed smooth mucosa, devoid of nodules in all patients. Nodular lesions appeared just beyond the apex of the bulb and were prominently seen in the second and third part of the duodenum. Nodular lesions were graded as grade 1 in 4 patients, grade 2 in 10 patients, grade 3 in 20 patients and grade 4 in 6 patients (mean ± 1SD grade 2.7 ± 1.2). All patients showed distensible duodenal lumen and none showed luminal narrowing or stricture or stasis or ulcerations. Examination of stomach showed no endoscopic abnormality in 8 patients; linear erythematous antral gastritis in 20 patients; exudative fundic gastritis in 6 patients and atrophic gastritis in 4 patients. Two patients had diffuse ulcerative nodular lesions limited to the antrum. None of the patient had pyloric or duodenal ulcer. RUT was positive in all patients and *H. pylori *were seen in gastric biopsies in all patients. Density of *H. pylori *was moderate in 6 patients and heavy in 34 patients. Histology of gastric biopsies revealed chronic superficial gastritis in 24 patients, and chronic atrophic gastritis with intestinal metaplasia in 14 patients. Two patients with diffuse ulcerative nodular disease of antrum showed histologic features of low grade MALT lymphoma. Duodenal biopsies showed mature nodes of lymphoid follicles in lamina propria (Figure 2). These lymphoid collections showed presence of highly reactive germinal centres. In addition, lamina propria showed mild increase in lympho-mononuclear cells. *H. pylori *were infrequently seen on the duodenal mucosa and in small numbers. Duodenal biopsies lacked features of coeliac sprue namely blunted villi, increased crypt depth, increased crypt villous ratio and epithelial cell lymphocytosis. Immunohistochemistry showed polyclonality of the cellular infiltrate which excludes possibility of duodenal lymphoma. There were no viral inclusions or *Giardia lamblia *seen in the tissue specimens.

**Figure 3:**
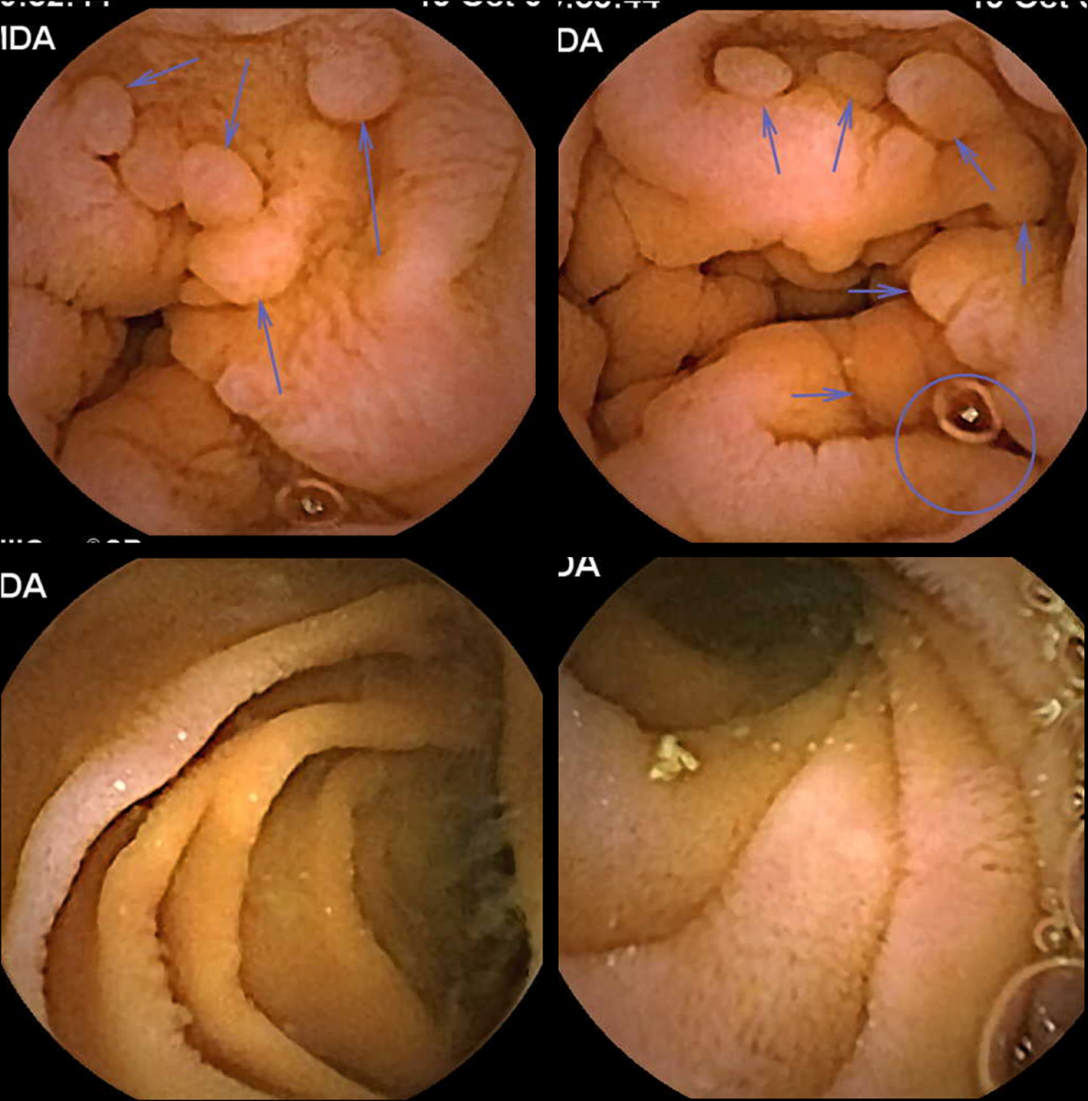
**Diffuse Duodenal Nodular Lymphoid Hyperplasia**. Video Capsule Endoscopy composite images. Top 2 images: Second and third part duodenum shows carpeting of the mucosa with nodular lesions. Bottom 2 images. Jejunum and ileum showed normal appearing mucosa without any nodular lesions.

Video capsule endoscopy showed nodular lesions in the postbulbar duodenum, second and third part of duodenum (Figure 3). There was marked reduction in the number of nodular lesions around the duodeno-jejunal junction. There were no nodular lesions seen in the jejunum and ileum. Colonoscopy and ileoscopy was normal in all cases and biopsies of the terminal ileum did not reveal nodular lymphoid hyperplasia.

**Figure 4:**
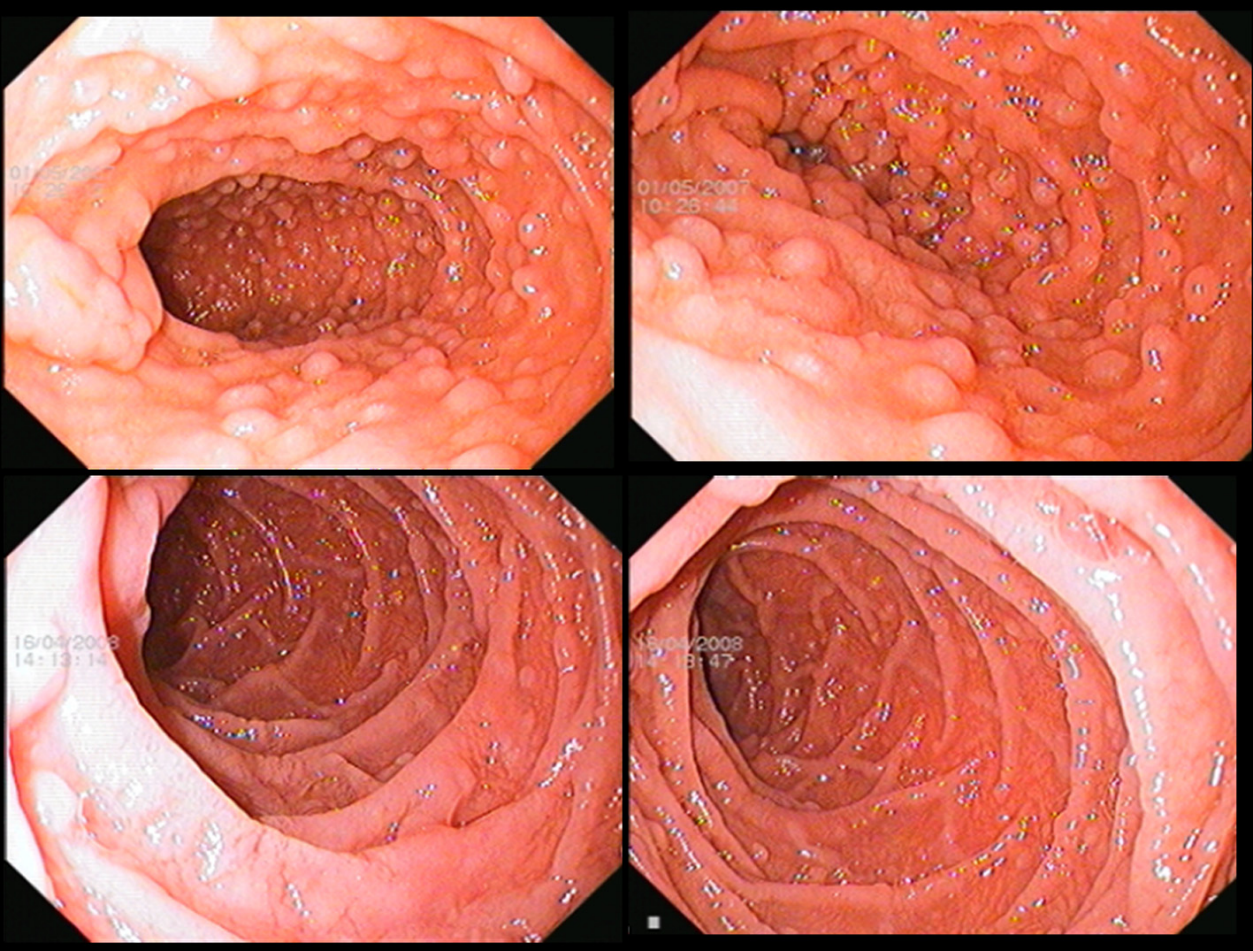
**Diffuse Duodenal Nodular Lymphoid Hyperplasia**. Duodenal composite images showing effect of *H. pylori *eradication. 25 year woman presented with recurrent epigastric pain, vomiting and weight loss of 10 kg over past 2 years. Immunoglobulin levels were within normal limits [IgG 1460 mg/dl (normal 700-1600 mg/dl); IgM. 190 mg/dl (normal = 40-230 mg/dl); IgA 230 mg/dl (normal = 70-400 mg/dl)]. Patient had *H. pylori *infection with severe fundic exudative gastritis. Top 2 images: Second part of duodenum was carpeted with numerous nodular lesions 3 to 5 mm in size, scored as grade 3 nodular disease. Patient received *H. pylori *sequential therapy and ^14^C UBT 6 weeks after therapy showed eradication of infection. Bottom 2 images. Follow up duodenoscopic images at one year showed near complete resolution of the nodular lesions (scored as grade 0). Repeat duodenal biopsies failed to show nodular lymphoid follicles.

**Figure 5:**
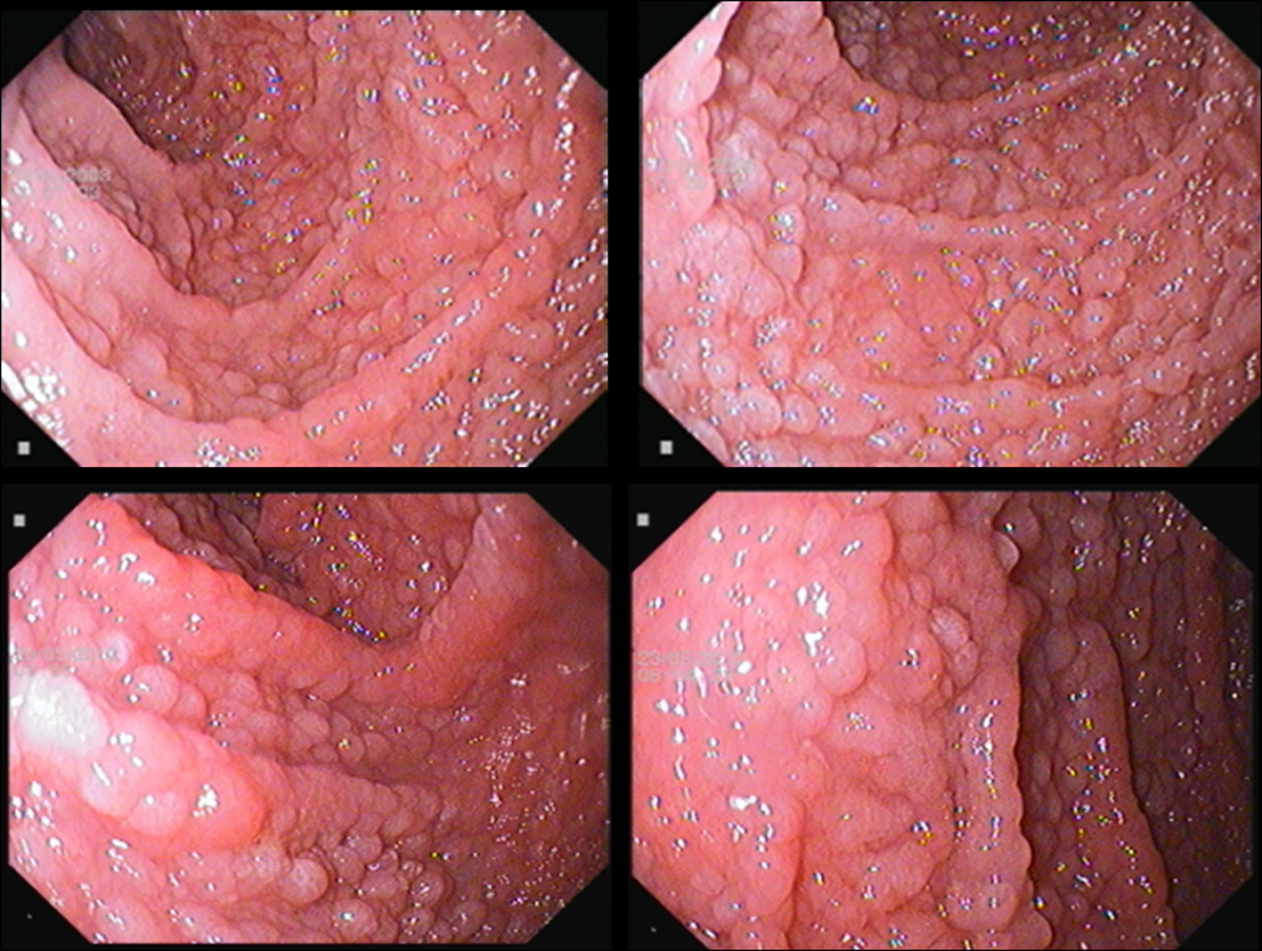
**Diffuse Duodenal Nodular Lymphoid Hyperplasia**. Duodenal composite images. Progression of disease. 40 year women presented with epigastric pain, vomiting, weight loss and recurrent diarrhoea. IgA levels were elevated [IgG 1400 mg/dl (normal 700-1600 mg/dl); IgM. 220 mg/dl (normal = 40-230 mg/dl); IgA 540 mg/dl (normal = 70-400 mg/dl)]. Patient had *H. pylori *related erythematous antral gastritis. Patient received *H. pylori *eradication sequential therapy followed by Levofloxacin/Doxycycline based second line eradication therapy and ^14^C UBT showed resistant *H. pylori *infection. Top 2 images. Duodenal mucosa showed diffuse infiltration with nodules of 3 to 5 mm size, scored as grade 3 nodular disease. Bottom 2 images at 2 years follow up. There was significant increase in size (>5 mm) and density of the nodular lesions. *Kerckring's folds *showed infiltration and focal thickening by nodular disease, scored as grade 4 nodular disease. Biopsies showed nodular lymphoid follicles and infiltrate showed polyclonality of the lymphocytes.

All patients received sequential antibiotic therapy. 14C UBT, done 4 to 6 weeks following antibiotic therapy, revealed eradication of *H. pylori *infection in 22 patients. Another 4 patients had *H. pylori *eradication following second line therapy. Remaining 14 patients had persistent *H. pylori *infection. Abdominal pain and vomiting showed improvement in 14 of the 26 patients in whom H pylori had been eradicated (p < 0.001) (Table 2). In contrast only 3 out of 14 patients with persistent *H. pylori *infection had improvement in symptoms (p = 0.22). Follow up duodenoscopies in 26 patients with *H. pylori *eradicated showed significant reduction of duodenal nodular lesions score (2.69 ± 0.79 to 1.50 ± 1.10; p < 0.001). Nodular lesions showed complete endoscopic and histological resolution in 5 patients and significant resolution in remaining 21 patients (Figure 4). Two patients with low grade MALT lymphoma showed endoscopic and histologic resolution of the disease, after *H. pylori *eradication. Patients with resistant *H. pylori *infection showed no significant reduction of nodular lesions score (2.71 ± 0.96 to 2.64 ± 1.15; p = 0.58). Nodules regressed in 2 patients, showed no interval change in 10 patients and progressed in 2 patients (Figure 5). Histology of nodules which had progressed over the follow up continued to show polyclonality and there was no suggestion of these nodules evolving in to lymphoma.

**Table 2:** The effects of *H. pylori *eradication therapy on 40 patients with diffuse duodenal nodular hyperplasia.

Disease severity	All patients (n = 40)			Eradicated group (n = 26)			Not eradicated group (n = 14)		
	H. pylori therapy		P value	H. pylori therapy		P value	H. pylori therapy		P value
	Before	After		Before	After		Before	After	
Persistent symptoms	40	23	<0.001a	26	12	<0.001a	14	11	0.22a
Grade of nodular disease									
Grade 0	0	5	0.005b	0	5	0.002b	0	0	0.91b
Grade 1	4	12		2	9		2	3	
Grade 2	10	11		7	7		3	3	
Grade 3	20	10		14	4		6	4	
Grade 4	6	2		3	1		3	4	
Mean ± 1SD Score	2.70 ± 0.84	1.83 ± 1.06	<0.001c	2.69 ± 0.79	1.50 ± 1.10	<0.001c	2.71 ± 0.96	2.64 ± 1.15	0.58c

a = Fisher's exact test 2 × 2 table; b = Fisher's exact test rxc table; c = students t test.

## Discussion

NLH of the gastrointestinal tract is a rare disorder, often reported with immune deficiency disorders and/or recurrent giardiasis [11-13,15,17-19]. The disease may be localized to a segment or may affect longer segments of bowel [13]. In contrast to reported disease in literature, the cohort of patients described in this study had significant differences. Firstly the disease was often encountered and report of 40 cases from one centre in 5 years period is a testimony to that. Second the disease involvement was limited to postbulbar duodenum, second and third part and duodeno-jejunal junction. Duodenal bulb was spared and there was no involvement of jejunum and ileum. Third none of the patients included in this study had immune deficiency or giardiasis. There are several reports of large cohort of patients similar to described by us from Mexico [16,29]. However, around half of such patients had hypogammaglobinemia.

Diarrhea and weight loss secondary to malabsorption has been the dominant symptom of NLH of small bowel as reported in the literature [1,15,30,31]. Malabsorption is a common symptom in patients with immune deficiency with or without superadded recurrent giardiasis [32]. In contrast our patients with DDNLH presented with epigastric pain and vomiting, clinically suggesting gastric stasis and obstruction. Only 6 of our patients presented with diarrhea which may suggest co-existent malabsorption. Weight loss, gastric symptoms, iron lack anemia, and hypoalbuminemia in our patients were mostly caused by selective and dominant involvement of the duodenal mucosa [31].

The pathogenesis of nodular lymphoid hyperplasia has been a matter of debate for long. Histology of these lesions demonstrates hyperplasic lymphoid follicles with mitotically active germinal centres. In immune deficiency states, the lymphoid hyperplasia is likely the result of an accumulation of plasma-cell precursors due to maturational defect in the development of B-lymphocytes [2,29]. These cells attempt to compensate for functionally inadequate intestinal lymphoid function. Bacterial contamination of small intestines is often mentioned as an etiological factor for NLH [16]. This is supported by regression of nodules following oral antibiotic therapy in some cases [32]. Some investigators suggest that coeliac disease may be associated with NLH [1]. However, NLH may occur in a whole spectrum of disorders without any abnormalities in immunoglobulins [33-35]. It is believed that NLH in absence of immune deficiency disorders may be related to immune stimulation of the gut lymphoid tissue.

*H. pylori *infection is etiologically associated with a number of gastroduodenal disorders. Acute infection causes neutrophilic gastritis with transient hypochlorhydria and subjects complain of epigastric pain and nausea [36]. Chronic infection causes a wide variety of gastritis including chronic superficial gastritis, nodular gastritis and chronic atrophic corpus gastritis with metaplasia [37,38]. *H. pylori *infection is strongly associated with peptic ulceration of duodenum and stomach [39,40]. Chronic corpus atrophic gastritis with intestinal metaplasia caused by *H. pylori *infection is an initiating event in most cases of intestinal type adenocarcinomas stomach. In fact *H. pylori *infection is associated with both diffuse-type and intestinal-type gastric adenocarcinoma [40,41]. Another entity which is etiologically related to *H. pylori *infection is gastric MALT lymphoma. The disease evolves through *H. Pylori *gastritis with mucosa associated lymphoid tissue (MALT), lymphoepithelial lesions, low grade B cell lymphoma and finally diffuse large B cell lymphoma [42-44]. There are no published reports of association of diffuse duodenal nodular lymphoid hyperplasia (DDNLH) with *H. pylori *infection.

A number of findings strongly pointed to *H. pylori *to be etiologically associated with DDNLH in our patients. All patients were infected with *H. pylori *infection. Patients in whom *H. pylori *were eradicated showed significant clinical response and regression/resolution of duodenal events. In contrast patients in whom *H. pylori *could not be eradicated showed persistence of clinical symptoms and persistence of duodenal nodular lesions. A number of earlier studies have suggested *H. pylori *as a possible cause of NLH [16,29], but none has followed this lead and none has substantiated this association.

What could be the pathogenesis of DDNLH in our patients? All patients had heavy *H. pylori *infection with advanced changes in the stomach as evidenced by gastroscopic and histological findings. In fact 2 of our patients had low grade MALT lymphoma. However, the duodenal changes seen were not as a result of direct involvement by *H. pylori*, as the organisms were not consistently present in the duodenal biopsies. A number of nongastrointestinal tract diseases are possibly associated with *H. pylori *infection. Many of these associations are suggested to be related to the effects of *H. pylori *on coagulation and markers of systemic inflammation. We believe the duodenal lesions were as a result of immune stimulation of prolonged and heavy *H. pylori *infection [45]. This was supported by elevated immunoglobulins in a number of patients in our series. Some extragastric disease states are particularly associated with H. *pylori *CagA-positive infections [46,47]. DDNLH seen in our community may be related to high prevalence of such *H. pylori *infections in our community and this need to be explored further. We believe careful examination of post-bulbar and second part of duodenum at EGD and liberal use of duodenal biopsies in patients with heavy *H. pylori *infection in tropical countries is needed to define the impact of this disease.

Two of our patients who failed to eradicate *H. pylori *infection had disease progression. However, biopsies showed prominent lymphoid follicles with active germinal centres located in the mucosa and there was no suspicion of disease evolving in to lymphoma. NLH has special relationship with lymphoma [7-9]. The disease needs to be differentiated from lymphoma. The presence of highly reactive germinal centres, numerous cell types, prominent vascularity, and polyclonality as determined immunohistochemically are the most important features in the differential diagnosis with lymphoma. Lymphoid hyperplasia may be differentiated from follicular lymphoma presenting as lymphomatous polyposis by Bcl-2 immunostaining of follicular germinal centres. NLH may be a manifestation of extraintestinal lymphoma and disease regresses after extraintestinal lymphoma undergoes remission under chemotherapy [48]. May patients with gastrointestinal lymphoma may present with NLH. Moreover, studies have shown that NLH itself may evolve in to lymphoma on long term follow up. The study period in our patients was not enough to define whether disease can evolve in to lymphoma. Long term follow up of these patients needs to be done to evaluate the malignant potential of this entity.

## Conclusions

In summary, we report on 40 patients with DDNLH. Patients presented with intractable dyspepsia and esophagogastroduodenoscopy showed diffuse nodular lesions of varying grades involving postbulbar duodenum. None of the patients had immunoglobulin deficiency or small intestine bacterial overgrowth or positive IgA endomysial antibodies. All patients were infected with *H. pylori *infection. Sequential antibiotic therapy eradicated *H. pylori *infection in 26 patients. Follow up duodenoscopies in these patients showed significant reduction of duodenal nodular lesions score. Fourteen patients with resistant *H. pylori *infection showed no significant reduction of nodular lesions score. We believe DDNLH in our patients was etiologically related to *H. pylori *infection.

## Competing interests

The authors declare that they have no competing interests.

## Authors' contributions

Contribution by individual authors:

MSK 1 conceived the study; participated in the design of the study; reviewed pathology and performed the statistical work. NSK participated in the design of the study; performed the blind scoring of the endoscopic findings and helped drafting the manuscript. MSK 3 participated in the design of the study; performed the blind scoring of endoscopic findings; and drafted the manuscript. All authors read and approved the final manuscript.

## Authors' information

1. Mehnaaz S Khuroo, MBBS, MD (Pathology), Lecturer, Department of Pathology, Government Medical college, Srinagar, Kashmir, India; Formerly Consultant Pathology, Jawahir Lal Nehru Memorial (JLMN) Hospital Rainawari, Srinagar, Kashmir, India. E-mail: mkhuroo@yahoo.com

2. Naira S Khuroo, MBBS, FIMR (KFSHRC Riyadh), Consultant Radiology, Digestive Diseases Centre, Srinagar, Kashmir, India. E-mail: naira_sultan@yahoo.com

3. Mohammad S Khuroo, MBBS,  MD, DM, FRCP (Edin), FACP, Master American College of Physicians (MACP, Emeritus), Consultant Gastroenterology, Digestive Diseases Centre, Srinagar, Kashmir, India. E-mail: khuroo@yahoo.com; visit at: http://www.drkhuroo.com

## Pre-publication history

The pre-publication history for this paper can be accessed here:

http://www.biomedcentral.com/1471-230X/11/36/prepub
